# Weight loss and self-perceived quality of life following laparoscopic Roux-en-Y gastric bypass: is it important?

**DOI:** 10.1590/0102-67202025000047e1916

**Published:** 2026-01-09

**Authors:** Gustavo Rodrigues Alves CASTRO, Laís Ducatti MACEDO, João Victor Vecchi FERRI, Isabella Benitez VULCANIS, Diancarlos Pereira de ANDRADE, José Alfredo SADOWSKI, Eduardo Lemos de Souza BASTOS, João Caetano Dallegrave MARCHESINI

**Affiliations:** 1 Faculdades Pequeno Príncipe – Curitiba (PR), Brazil.; 2 Hospital São Marcelino Champagnat – Curitiba (PR), Brazil.; 3 Santa Casa de Misericórdia de Marília – Marília, (SP), Brazil.

**Keywords:** Bariatric Surgery, Gastric Bypass, Anastomosis, Roux-en-Y, Laparoscopy, Quality of Life, Weight Loss, Cirurgia Bariátrica, Derivação Gástrica, Anastomose em-Y de Roux, Laparoscopia, Qualidade de Vida, Redução de Peso

## Abstract

**Background:**

Weight loss (WL) is the most commonly used datum to measure the results of metabolic and bariatric surgery. The amount of WL is generally directly and proportionally associated with the improvement in quality of life (QoL), as the greater the former, the greater the perception of well-being.

**Aims:**

To assess the relationship between the amount of weight lost after laparoscopic Roux-en-Y gastric bypass (LRYGB) and self-perceived improvement in quality of life (QoL).

**Methods:**

The medical records of patients who underwent LRYGB between January 2017 and December 2019 with a minimum follow-up of 3 years were analyzed. The data obtained in the subgroups made up according to percentage of total weight loss (%TWL), age, and time elapsed since surgery were compared with the self-perceived QoL by the Short Form Survey 36 (SF-36) questionnaire.

**Results:**

A total of 95 individuals (71.6% women) with an average age of 45 years and an average postoperative (PO) follow-up of 61.1 months were enrolled. The mean pre- and postoperative weight was 114 kg and 73.4 kg, respectively, and the mean %TWL was 35.6%. According to the comparison between the data from the medical records and the self-perceived QoL assessment, D1 (physical functioning) was the best scoring domain, while D3 (pain) was the worst. There was a significant improvement of the D4 (general health) domain in patients with %TWL greater than 30% (p<0.05), D7 (role emotional), and D8 (mental health) domains in patients older than 45 years (p<0.05) and better results in D7 (role emotional) domain in patients over 5 years after surgery (p<0.05).

**Conclusions:**

Greater weight loss and age and longer time after surgery showed important self-perceived improvement in QoL after LRYGB in some assessment domains, both physical and emotional.

## ARTICLE HIGHLIGHTS

- The amount of weight loss is generally directly and proportionally associated with the improvement in quality of life (QoL);
- Nonsignificant reductions in body weight may also be enough to increase the QoL in physiological, psychological, social, and economic aspects;
- Laparoscopic Roux-en-Y gastric bypass (LRYGB) can have a direct relationship with significant positive impacts on health-related self-perceived QoL, sustained in the long-term;
- Greater weight loss and age and longer time after surgery showed important self-perceived improvement in QoL after LRYGB in physical and emotional domains.

## CENTRAL MESSAGE

Weight loss (WL) is the most commonly used datum to measure the results of metabolic and bariatric surgery. The amount of WL is generally directly and proportionally associated with the improvement in quality of life (QoL), as the greater the former, the greater the perception of well-being. However, improvements in QoL may also not be linearly related to the amount of weight lost, as nonsignificant reductions in body weight may already be sufficient to increase QoL in physiological, psychological, social, and economic aspects. Furthermore, QoL seems to be one of the main targets of patients seeking bariatric procedures, which is not always properly valued.

## PERSPECTIVES

In the present study, we demonstrate that the amount of weight lost provided by laparoscopic Roux-en-Y gastric bypass can have a direct relationship with significant positive impacts on health-related self-perceived QoL, both physical and emotional. Moreover, these effects appear to be sustained in the long term, especially in older individuals.

## VISUAL ABSTRACT

**Figure:**
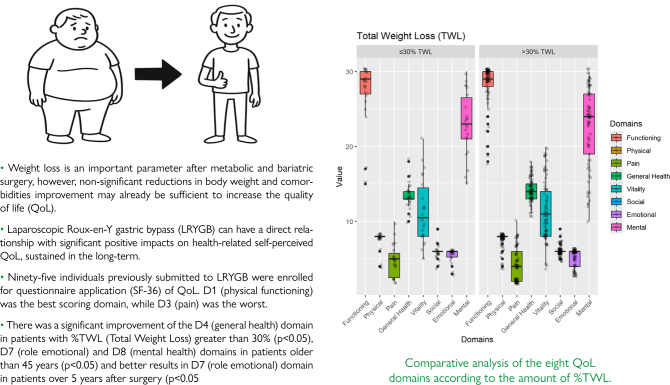

## RESUMO

**Racional:**

A perda de peso (PP) é o dado mais comumente utilizado para mensurar os resultados da cirurgia bariátrica e metabólica. A quantidade de PP geralmente está direta e proporcionalmente associada à melhora da qualidade de vida (QV), visto que quanto maior a primeira, maior a percepção de bem-estar.

**Objetivo:**

Avaliar a relação entre a quantidade de peso perdido após *bypass* gástrico laparoscópico em Y de Roux (BGYR) e a melhora autopercebida na qualidade de vida (QV).

**Métodos:**

Foram analisados os prontuários médicos de pacientes submetidos a BGYR entre janeiro de 2017 e dezembro de 2019 com um acompanhamento mínimo de 3 anos. Os dados obtidos nos subgrupos compostos de acordo com a porcentagem média de perda de peso total (%PPT), idade e tempo decorrido desde a cirurgia, foram comparados com a QoL autopercebida por meio do questionário *Short Form Survey* 36 (SF-36).

**Resultados:**

95 indivíduos (71,6% feminino) com idade média de 45 anos e acompanhamento pós-operatório médio de 61,1 meses foram analisados. Os pesos médios pré- e pós-operatórios foram de 114 kg e 73,4 kg, respectivamente, e a %PPT média foi de 35,6%. A comparação entre os dados dos prontuários e a avaliação da autopercepção da QoL mostrou que D1 (capacidade funcional) foi o domínio com melhor pontuação, enquanto D3 (dor) foi o pior. Houve melhora significativa do domínio D4 (estado geral de saúde) em pacientes com %PPT maior que 30% (p<0,05), domínios D7 (aspectos emocionais) e D8 (saúde mental) em pacientes com mais de 45 anos (p<0,05), além de melhores resultados no domínio D7 em pacientes com mais de 5 anos de cirurgia.

**Conclusões:**

A maior perda de peso e a idade, e maior tempo após a cirurgia mostraram melhora autopercebida importante na QV após BGYR em alguns domínios de avaliação, tanto físicos quanto emocionais.

## INTRODUCTION

Weight loss (WL) is the most commonly used datum to measure the results of metabolic and bariatric surgery (MBS). According to the Position Statement of the Brazilian Society of Bariatric and Metabolic Surgery (SBCBM), at least 20% total WL must be achieved to be considered successful^2^, but a universally accepted consensus is still uncertain. Conversely, other parameters can also measure the outcome of a bariatric procedure such as quality of life (QoL) and improvement or remission of comorbidities^1,4,12^.

QoL is not very easy to accurately measure, as it is based on the patient’s subjective perception of the overall quality of their life after the bariatric procedure^1^. Even so, some tools have already been validated in the literature and are frequently used for this purpose^6,18^. One of them is the Short Form Survey 36 (SF-36) questionnaire, a widely-used and validated assessment method for both surgical and clinical conditions, providing important data on topics related to QoL^7,14^. It consists of eight domains, evaluating physical and mental aspects, being useful in comparing general and specific populations. Additionally, it can estimate the relative burden of different diseases and highlight the health benefits produced by a wide range of treatments^11,13^.

In the real world, the amount of WL is generally directly and proportionally associated with improvements in QoL, as the greater the former, the greater the perception of well-being^8^. However, improvements in QoL may also not be linearly related to the amount of weight lost, as nonsignificant reductions in body weight may already be sufficient to increase QoL in physiological, psychological, social, and economic aspects. Furthermore, QoL seems to be one of the main targets of patients seeking bariatric procedures, which is not always properly valued by doctors.

Therefore, in the present study, we aim to evaluate the QoL of patients undergoing laparoscopic Roux-en-Y gastric bypass to assess whether there is any linear relationship between the percentage of total weight loss (%TWL) and improvements in QoL.

## METHODS

### Study design

This is a retrospective study, with a quantitative approach carried out through document analysis and questionnaire application.

### Study population

All patients who underwent MBS in a single center of excellence between January 2017 and December 2019 were considered eligible for the study. Inclusion criteria encompassed being over 18 years old, having undergone LRYGB surgery, and at least 3 years of follow-up. Exclusion criteria were applied to patients who underwent revisional LRYGB and patients who did not agree to answer the QoL assessment questionnaire, or did not fill it out correctly or completely.

### Data collection

The study was approved by the Research Ethics Committee of our Institution (number 6029928) and consisted of two stages. First, demographic data — such as age, sex, date of surgery, type of procedure, preoperative weight, weight at the last postoperative (PO) consultation, preoperative body mass index (BMI), and BMI at the last PO consultation — were retrieved from medical records. In the second stage, patients who met the inclusion criteria were contacted via telephone and invited to participate in the study. Two phone call attempts were considered before excluding non-responding patients. For those who have been successfully contacted and agreed to take part in the study, the SF-36 Medical Outcomes Study questionnaire was electronically sent along with the Informed Consent Form. Patients were asked to send back, also electronically, the QoL assessment instrument and the signed Consent Form within two weeks.

### Subgroups analyzed

Based on preoperative weight and the weight at the last PO consultation, the percentage of total weight loss (%TWL) was calculated using Equation 1:

[Preoperative weight – Current weight / Preoperative weight] x 100(1)

Subsequently, patients were divided into two groups according to %TWL at ≤30% and >30%. Furthermore, for comparative analysis with QoL, patients were divided according to age (≤45 and >45 years) and time elapsed since surgery (≤60 and >60 months).

### QoL assessment questionnaire

The questionnaire used for the analysis was the Short Form Survey 36 (SF-36), a generic health survey consisting of 36 questions used to measure self-reported QoL. It assesses various domains of physical and mental health and provides summary scores for physical and mental components. It is necessary to observe the relevant domains of the questionnaire and the score belonging to each of them individually, that is, a total of 8 domains, with 36 questions. Therefore, the proportion of scores is standardized, making the comparison between dimensions feasible^11,13^.

The domains covered are: D1 (Physical Functioning); D2 (Role Limitations due to Physical Aspects, or Role Physical); D3 (Pain); D4 (General Health); D5 (Vitality); D6 (Social Function); D7 (Role Limitations due to Emotional Problems, or Role Emotional); and D8 (Mental Health). The values of each domain are scored from 0 (zero) to 100 (one hundred), from worst to best. Individuals who agreed to participate received an anonymous link to respond to the questionnaire electronically.

### Primary end point

Assessment of the self-perceived QoL obtained through the application of the SF-36 according to %TWL with at least 3 years of follow-up.

### Secondary end point

Comparison of the self-perceived QoL obtained through the application of the SF-36 with age and time elapsed since surgery.

### Statistical analysis

The R program version 4.1.2 was used in all analyses. The descriptive analysis was carried out by checking quantities and percentages for categorical variables and descriptive measures (minimum, maximum, quartiles, mean, and standard deviation) for continuous variables. The Student’s t-test was used to compare the means of the variables on the Likert scale between two independent groups. If the calculated p-value is greater than the critical t-value for a given level of significance (p<0.05), the conclusion is that there is a significant difference between the means of the two samples.

## RESULTS

### Study population

Between January 2017 and December 2019, 536 patients with obesity underwent MBS at the study Hospital. Of these, 441 (82.2%) did not meet the inclusion criteria, were unreachable, refused to participate in the study, or did not adequately complete the QoL assessment instrument. Therefore, our final sample size comprised 95 (17.7%) patients undergoing primary LRYGB who adequately completed the QoL assessment instrument.

### Demographic data

Of the 95 patients included in the study, 68 (71.6%) were women and the average age of the entire sample was 45 years (ranging from 22 to 74). The mean preoperative weight was 114 kg (74–193) and mean PO weight was 73.4 kg (47–158). According to BMI data, there were preoperative and PO means of 40.3 kg/m^2^ (32–58.9) and 25.9 kg/m^2^ (18.6–48.2), respectively.

The mean %TWL was 35.6%. Regarding time elapsed from surgery to the moment of application of the QoL assessment instrument, the average was 61.1 months (45–81 months).

### Overall analysis

The best scored domain was D1 (Physical Functioning), with a maximum score of 90.7, followed by D2 (Role Physical) with 86.3, showing a greater functional capacity described by patients. Meanwhile, the minimum score was observed in the D3 domain (pain), with 23.58, highlighting it as a relevant negative factor following the procedure.

### Assessment of QoL according to %TWL

**Figure 1:**
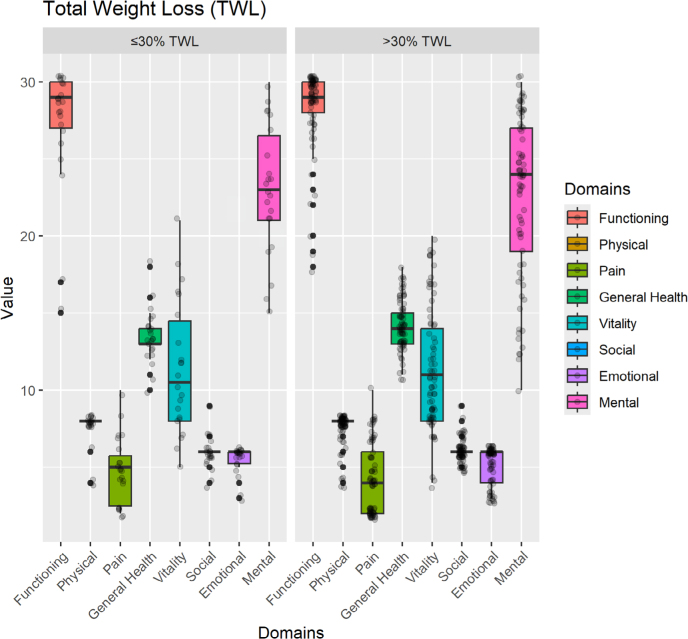
Comparative analysis of the eight quality of life domains according to the percentage of total weight loss.

As shown in Figure 1, individuals with %TWL greater than 30% reported better self-perception of their general health (D4 domain) (p<0.05). This finding allows us to infer that the greater the weight loss, the better the overall QoL after LRYGB surgery.

### Assessment of QoL according to age

**Figure 2:**
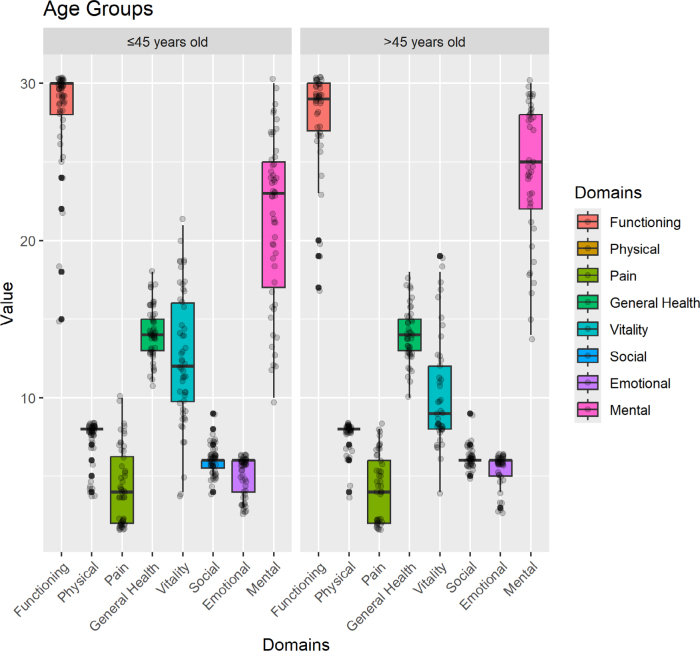
Comparison of qualify of life scores between age groups according to domains of the Short Form Survey 36 questionnaire.

We observed that the older group reported better scores in D7 (Role Emotional) and D8 (Mental Health) (p<0.05), showing a better performance than the younger group (Figure 2).

### Assessment of QoL according to PO period

**Figure 3:**
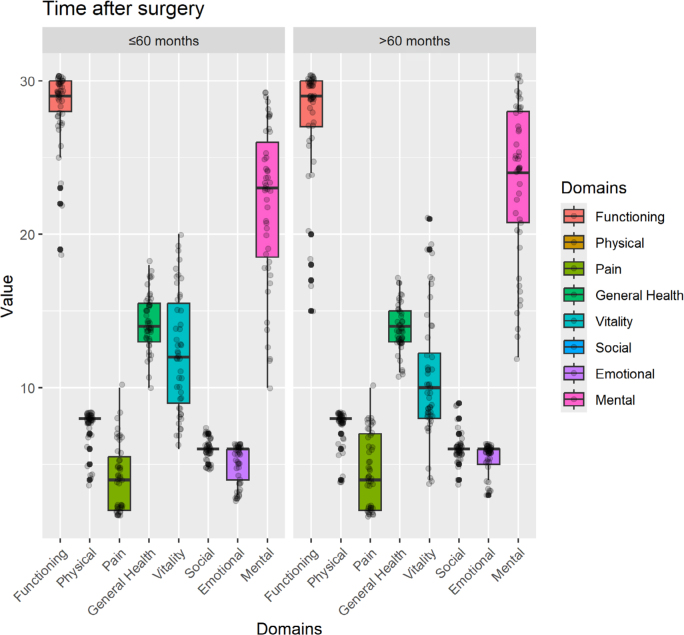
Comparison of quality of life scores between groups according to the time elapsed since surgery.

According to the time elapsed since surgery, the sample was divided into a group with less than 60 months of follow-up and another with over 60 months of follow-up; the latter showed better results in D7 (Emotional Role; p<0.05), as shown in Figure 3.

## DISCUSSION

In the present study, we verified that there may be a linear association between the amount of weight lost after LRYGB surgery and general self-perceived improvement in QoL. We also found that not only physical function improved, but also mental aspects, especially in the long term and in older patients.

In our study, the impact of WL after MBS on QoL was measured using the SF-36 questionnaire, which is composed of 36 items divided into eight domains^14^, used in various clinical settings to study the impact of chronic diseases on health-related QoL. Khawali et al.^7^ compared 41 patients with severe obesity on a waiting list for bariatric surgery with 84 patients undergoing MBS in a public health system. The group that had already undergone the procedure had higher scores in all domains of the SF-36 questionnaire than the preoperative group. Similarly, authors of a study encompassing 65 bariatric patients also used the SF-36 tool to assess QoL, and showed that QoL improved significantly after 1 year of MBS^12^.

In addition to the SF-36 questionnaire, there are other methods for assessing QoL in bariatric patients. The most popular is probably the Bariatric Analysis and Results Reporting System (BAROS) protocol^17^. Nonetheless, the BAROS protocol has body weight loss among its scores, namely percentage of excess weight loss (%EWL), making the dissociation of this variable from the others somewhat nebulous^16^.

Another tool that can also be used is the Moorehead-Ardelt Quality of Life Questionnaire II, which can be used independently or incorporated into the BAROS protocol^3,15,17^. This instrument assesses several pre- and PO comorbidities, such as hypertension, diabetes mellitus, peripheral vascular disease, dyslipidemia, sleep apnea, and infertility, as well as self-esteem, physical activities, social relationships, labor, and libido. However, it is not able to assess the QoL of patients with extreme obesity properly^12,15^.

In our study, physical, emotional and mental aspects were the best scored domains in the overall analysis, while pain was the worst. There are a wide variety of causes for pain after LRYGB surgery that may explain this lower score, including behavioral and nutritional disorders, functional motility disorders, marginal ulceration, and internal hernia. Although sometimes not clinically concerning, surgeons should be aware of this self-perception, as it may negatively impact QoL assessment.

Regarding the relationship between WL and self-assessment of QoL, our findings are as expected, considering that WL seems to be the main expectation of patients with obesity when undergoing bariatric procedures. Therefore, patients with greater WL tend to better evaluate their QoL, even minimizing some dysfunctions related to the nature of bariatric surgeries. We corroborate the findings of a systematic review whose authors evaluated the impact of WL on health-related QoL in overweight and obesity adults using the SF-36 and the Impact of Weight on Quality of Life-Lite (IWQOL-Lite) instruments. The authors provided evidence supporting that WL achieved by MBS can significantly improve QoL. Furthermore, sustained WL seems to be associated with maintenance of improvements in QoL, although these improvements were only significant for physical, rather than mental domains^10^.

As for the time elapsed since surgery, our study demonstrated a positive association with emotional aspects of the patients (D7 — Role Emotional), even in the long term after surgery. We corroborate other studies. Sierżantowicz et al.^19^ carried out a systematic review to analyze the health-related QoL changes in patients who underwent bariatric surgery with at least 9 years of follow-up. The authors observed that, despite the decrease in the QoL scores from the second year onwards, their values at 9–12 years were still significantly higher than at the baseline, showing that the positive effects of bariatric surgery on QoL can be sustained at the long term. The finding that the improvement in self-perceived QoL is more significant in the first years after bariatric surgery, but that it can also be sustained in the long term, is corroborated by another systematic review whose authors included 40 studies in a qualitative analysis. Interestingly, the researchers showed consistent results regardless of the instrument used to assess QoL^18^.

Furthermore, we demonstrated that older patients reported better scores in emotional and mental domains after the procedure, explained perhaps by maturity or differences between generations. Similarly, Gerber et al.^5^ found that older individuals (>60 years old), preoperatively, scored better on mental aspects than the entire cohort, and PO improvements in physical components and obesity problems were sustained in all age groups.

Undoubtedly, physical aspects seem to perform better than the psychological domains after MBS, whatever the QoL assessment tool. In a very long-term prospective cohort (12 years), patients undergoing gastric bypass significantly sustained their improvement in QoL in both weight domains of the IWQOL-Lite instrument and physical domains of the SF-36 instrument when compared with the non-surgical group, but this superiority was not sustained in terms of mental/psychological aspects^9^. Conversely, in our study, emotional and mental aspects actually improved, especially after 5 years of the procedure and in older patients. Perhaps our commitment to maintaining close psychological and, whenever necessary, psychiatric follow-up for all patients, regardless of mental illness diagnoses, may be the reason for this finding.

We are aware that our study has some limitations that could weaken the findings. First, we had significant missing data, despite our great efforts to contact enrolled patients and try to convince them to participate in the study. Unfortunately, many of them refused to fill out the questionnaire. Second, the cross-sectional design of the study has its inherent downsides preventing an evolutionary assessment. Third, we only included patients undergoing gastric bypass. Obviously, our findings cannot be applied to other types of bariatric procedures. Finally, the assessment of QoL by self-report is highly subjective and not always reliable, although questionnaire designs have already been validated.

Notwithstanding, according to our findings, we clearly verified positive impacts on the QoL of patients undergoing LRYGB surgery, mainly related to the physical and functional domains. Furthermore, we also showed interesting beneficial effects on mental and emotional aspects, sustained in the long term.

## CONCLUSIONS

In the present study, we demonstrate that the amount of weight lost provided by LRYGB can have a direct relationship with significant positive impacts on health-related self-perceived QoL, both physical and emotional. Moreover, these effects appear to be sustained in the long term, especially in older individuals.

## Data Availability

The information regarding the investigation, methodology and data analysis of the article is archived under the responsibility of the authors.
